# Comparing the risk factors of nephrolithiasis in Asian countries population: A systematic review and meta-analysis

**DOI:** 10.1080/20905998.2023.2254960

**Published:** 2023-10-16

**Authors:** Zulfikar Ali, Reginald Rustandi, Mohammad Sulchan, Ponco Birowo, Tri Indah Winarni

**Affiliations:** a Doctoral Study Program of Medical and Health Science, Universitas Diponegoro, Semarang, Indonesia; b Department of Urology, Faculty of Medicine Universitas Indonesia, Cipto Mangunkusumo Hospital, Jakarta, Indonesia; c Department of Nutrition, Faculty of Medicine, Universitas Diponegoro, Semarang, Indonesia; d Center for Biomedical Research (CEBIOR), Faculty of Medicine, Universitas Diponegoro, Semarang, Indonesia

**Keywords:** Urolithiasis, Asian, risk factor, kidney stone

## Abstract

This systematic review and meta-analysis aims to identify the main risk factors for nephrolithiasis in Asian populations, with comparisons to European and American populations. Using a comprehensive literature search across PubMed, Science Direct, and ResearchGate, in accordance with the Preferred Reporting Items of Systematic reviews and Meta-Analysis (PRISMA) guidelines, we synthesized data from 11 geographically diverse studies. Our findings reveal substantial population-specific differences in nephrolithiasis risk factors, particularly familial history, water consumption, and smoking patterns. In Asian populations, a 60% increase in risk was associated with a family history of nephrolithiasis. In the meantime, drinking sources also affected nephrolithiasis risk, with the consumption of boiled water being associated with a 25% increase in risk compared to consumption of bottled or mineral water. These findings highlight the importance of tailoring preventive strategies and treatments to specific risk factors, taking into account regional variations, and call for additional research to understand the complex interaction between genetic, environmental, and lifestyle factors in the development of nephrolithiasis.

## Introduction

Nephrolithiasis, more commonly known as kidney stones, is a prevailing health issue of significant concern affecting millions of people globally [1]. With its pervasive reach, the occurrence and characteristics of nephrolithiasis display noticeable variations across distinct geographic regions and populations. These disparities are observed not just in incidence rates but also extend to more intricate features like stone composition. For instance, a noteworthy difference is observed between developed and developing countries, where calcium-containing stones tend to be more prevalent in developed nations [2].

Such contrasts can likely be attributed to differences in dietary habits, driven by socioeconomic factors, access to specific food groups, cultural preferences, and other local conditions. Consequently, understanding these regional variations and associated risk factors can provide valuable insights into the underlying pathophysiology of nephrolithiasis. This knowledge could potentially guide the development of region-specific preventive measures and treatment strategies, thereby enhancing the effectiveness of interventions [1].

Several international studies have embarked on the journey to identify and understand the complex landscape of risk factors contributing to nephrolithiasis formation. A consensus has been reached to categorize the significant risk factors into four primary domains: dietary, genetic, environmental, and lifestyle. These categories encompass a multitude of specific elements including age, sex, race, daily habits, genetic predispositions, smoking habits, and various environmental exposures [3,4].

Nevertheless, studies exploring the risk of nephrolithiasis in Asian population is still scarce, and we find it important to perform pooled analysis from existing studies to bring together a large body of existing evidence, synthesizing the findings of multiple studies to provide a more comprehensive and reliable understanding of the risk factors associated with nephrolithiasis. This approach allows for a more robust analysis of the available data, potentially revealing patterns, trends, and associations that may not be evident from individual studies alone.

This systematic review aims to find out the most contributing risk factors of nephrolithiasis in Asian populations, with insight from European and American populations for comparison. By synthesizing the available evidence, this review seeks to identify the differences in the risk factors associated with the development of nephrolithiasis in the Asian population.

## Material and methods

A comprehensive review of the published scientific literature up to March 2023 was conducted across a variety of online sources, including PubMed, Science Direct, and ResearchGate with the following subject terms and keywords applied using the boolean operators: ‘nephrolithiasis’ or ‘kidney calculus’ or ‘kidney stone’ and ‘risk factors’ and ‘asian’. Studies were incorporated according to eligibility criteria and were assessed using the EndNote application for possible duplication.

### Study selection

Studies examining the risk factor of nephrolithiasis, published during the previous 20 years, study type of case control and cross-sectional, studies written in English, and study with Asian population were the criteria for inclusion in this study. Meanwhile our defined exclusion criteria of the study were unavailable full text, irrelevant results or outcomes, and full text not in English.

**Figure 1.**
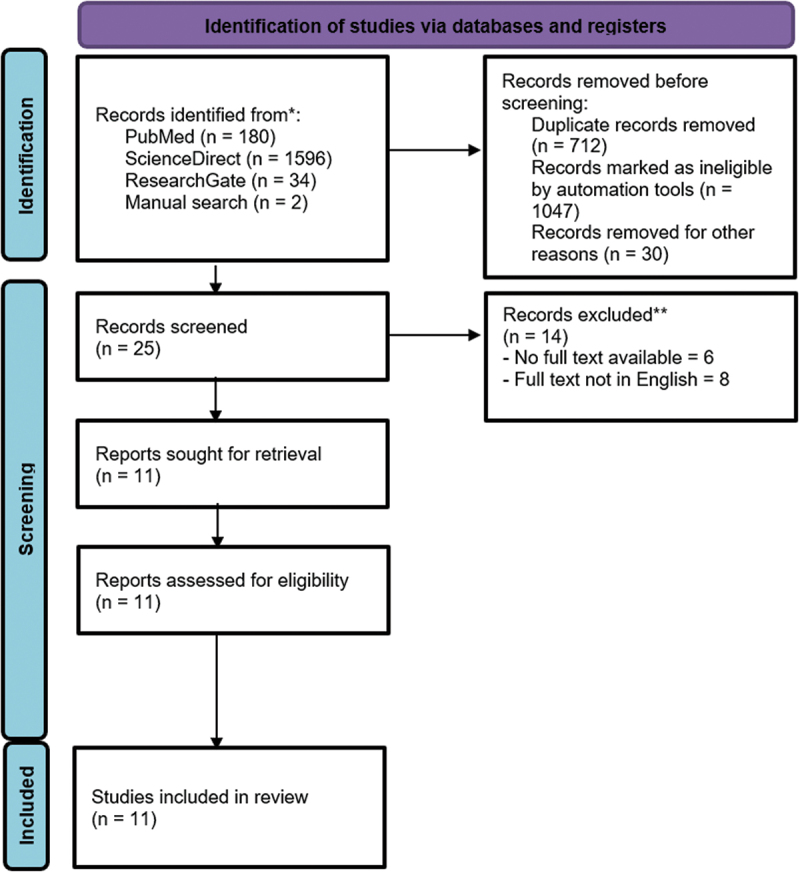
PRISMA flow diagram.

We applied PRISMA guidelines (preferred reporting items for systematic reviews and meta-analyses) to aid the process of study selection (Figure 1). After removing the duplicates, a total of 25 studies were evaluated. Of those 25 studies, full text was not available for four studies and eight studies were not in English. From the remaining studies, two were not available for study assessment. Selected 11 articles were then included for analysis and evaluated for their quality using the Newcastle-Ottawa scale (Tables 2 and 3).

### Data collection and statistical analysis

Study heterogeneity was evaluated using the I^2^ test. Heterogeneity above 50% was considered significant; therefore, a random-effect model would be designated for analysis. On the contrary, a fixed-effect model would be chosen when the heterogeneity proves to be insignificant. Analyses were carried out using statistical software Review Manager 5.4 by Cochrane Collaboration, Oxford, United Kingdom.

## Results

**Figure 2.**
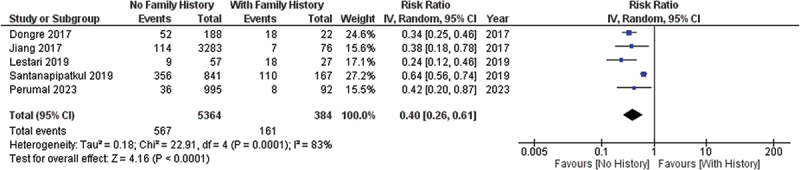
Forest plot of family history effect to the risk of nephrolithiasis.

Quantitative analysis as shown in forest plot in Figure 2 from four studies in Asian populations implies that having a family with history of nephrolithiasis is one of the risk factors to develop nephrolithiasis in an individual. Pooled analysis showed an increase of 60% risk of nephrolithiasis in individuals with a family history of nephrolithiasis, compared to those who do not have family history of nephrolithiasis.

**Figure 3.**
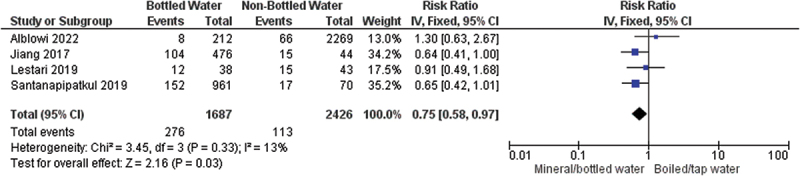
Forest plot of water source for drinking impact to the risk of nephrolithiasis.

Drinking habit was another factor that identified as a risk factor for, but the odds ratio and source of drinking water varied between studies. A pooled analysis from four studies in Asian population showed that drinking habits from boiled water have a significant impact in increasing the risk of nephrolithiasis by 25% compared to those who drink bottled or mineral water, as shown in Figure 3.

Aside from mineral water and boiled water, other sources of water from another study were varied, such as ground water, rain water, and filtered water. However, since water source is varied between studies, not all sources of water could be analyzed quantitatively for their association with the risk of nephrolithiasis as seen in Table 1.

**Table 1.** Summary of comparative studies included in meta-analysis.

Author, year	Country	Type of study	Sample size	Range/mean of sample age	Outcome
**Southeastern Asian Studies**					
Matsuba T, 2005^4^	Laos	Case control	221: 114 cases, 97 controls	N/A	Cigarette smoking: OR 6.12 (*p* < 0.05) Well as origin of drinking water: OR 1.93 (*p* < 0.05)
Lestari DA, 2019^5^	East Java, Indonesia	Case control	81: 27 cases, 54 controls	N/A	Family history: OR 0.09 (0.03–0.27) Male gender: OR 1.60 (0.05–4.70) Calcium source consumption: OR 0.85 (0.18–3.85)
Santanapipatkul K, 2019^6^	Loei Province, Thailand	Case control	161 cases, 170 controls	54.5 ± 12.8	Source of drinking water: • Boiled water: OR 0.18 (0.05–0.72) • Bottled water: OR 0.25 (0.13–0.50) • Rainy water: OR 3.32 (*p* < 0.001)
Perumal KJ, 2023^7^	Malaysia	Cross-sectional	1087: 486 males, 601 females	≥18 years old	Family history: OR 2.56 (1.14–5.56) Prefer salty food: 2.56 (1.37–4.76)
**Southern Asian Studies**					
Dongre, 2017^8^	Puducherry, India	Case-control	210; 70 cases, 140 controls	N/A	Family history: OR 16.98 (3.02–95.25) Source of drinking water: Ground water OR 1.13 (0.61–2.09) compared to river water
Singh, 2023^9^	New Delhi, India	Cross-sectional	60; 42 males, 18 females	N/A	Physicological stress: OR 2.98 (1.04–8.52)
Khan, 2022^10^	Rawalpindi, Pakistan	Cross-sectional	143; 81 males, 62 females	Mean±SD: 16.34 ± 17.02	Tap water as drinking water 56.64%
**Eastern Asian Studies**					
Jiang, 2017^3^	Beijing, China	Cross-sectional	3.350; 1.091 males, 2.259 females	Mean±SD: 48.97 ± 17.02	Male gender: OR 102.681 (1.062–9925.797)
**Western Asian Studies**					
Alblowi, 2022^11^	Jeddah and Riyadh, Saudi Arabia	Cross-sectional	1031 participants	≥18 years old	Daily consumption of black tea: OR 2.16 (1.42–3.29) Daily consumption of energy drink: OR 10.18 (4.0–25.92)
Moftakhar, 2022^12^	Iran	Cross-sectional	1.663; 4.719 males, 5.944 females	Mean±SD: 52.15 ± 8.22	Male gender: OR 1.1 (0.96–1.26)
Safdar, 2021^13^	Saudi Arabia	Cross-sectional	580; 251 males, 329 females	Mean±SD 27.5 ± 1.8	Age between 21 and 25 years, and older than 47 years old *p* < 0.005

**Table 2.** Summary of case–control study quality assessment using Newcastle-Ottawa scale.

Study author	Selection				Comparability	Exposure			Total score	Overall grade
	Case definition	Represent ativeness	Control selection	Control definition		Ascertainment	Methods	Non-response rate		
Matsuba 2005	★	★	★	★	★	★	★	★	7	Good
Lestari 2019	★	★		★	★		★	★	6	Good
Santana-Pipatkul 2019	★	★	★	★	★★	★	★	★	9	Good
Dongre 2017	★	★		★	★	★	★	★	7	Good

Stars indicate the rating according to the Newcastle-Ottawa Scale.

Good quality : 3 or 4 stars in selection domain AND 1 or 2 stars in comparability domain AND 2 or 3 stars in exposure domain.

Fair quality : 2 stars in selection domain AND 1 or 2 stars in comparability domain AND 2 or 3 stars in exposure domain.

Poor quality : 0 or 1 star in selection domain OR 0 stars in comparability domain OR 0 or 1 stars in exposure domain.

**Table 3.** Summary of cross-sectional quality assessment using adapted Newcastle-Ottawa scale for cross-sectional studies.

Study author	Selection			Comparability	Outcome		Total score	Risk of bias
	Represent ativeness	Sample size	Non-included subjects		Assessment	Statistical test		
Perumal 2023	★	★		★★	★	★	6	Medium
Singh 2023	★		★	★★	★★	★	7	Low
Khan 2022	★		★	★	★★		5	High
Jiang 2017	★	★	★	★★	★★	★	8	Low
Alblowi 2022	★	★		★★	★★	★	7	Low
Moftakhar 2022	★	★	★	★★	★★	★	8	Low
Safdar 2021	★	★	★	★	★		5	High

Stars indicate the rating according to the Newcastle-Ottawa Scale.

Low risk of bias : Studies that scored a total of 8 or 7 points were considered to have a low risk of bias.

Medium risk of bias : 6 points were considered to have a medium risk of bias.

High risk of bias : 5 points or less were considered to have a high risk of bias.

Another factor that was also mentioned to be a risk factor in those studies was consumption of some medications such as Losartan and Statins, as stated in a study from Landgren et al. [5] Aside from that, external factors such as psychological and psychosocial stress were also mentioned as risk factors. However, the association was not found in other studies.

## Discussion

Known risk factors for urolithiasis include intrinsic factors (such as age, gender, race, and family history) and extrinsic factors (such as dietary practices, water intake, and geographic location). In accordance with the findings of Santanapipatkul et al. [6] our study highlights the substantial effect of family history on an individual’s risk for developing nephrolithiasis. Family history is a combination of both internal and external factors, as family history may arise from both genetic predispositions, while it may also arise from similar dietary intake and water sources [7]. This suggests the presence of a genetic component, implying that future research may find it useful to investigate potential genetic markers and predispositions.

Another main factor that our research examines is the intricate relationship between water consumption and nephrolithiasis. The apparent increase in nephrolithiasis risk associated with the consumption of boiled water is a particularly intriguing finding. This contradicts prevalent theories, such as those proposing that a known risk factor, a high calcium concentration in well water, can be mitigated by simmering water to precipitate calcium carbonate compounds [8]. Due to its higher calcium content, drinking unboiled well water would increase the risk of nephrolithiasis, according to this reasoning. In contrast, our findings indicate that drinking boiled water may increase the risk of nephrolithiasis when compared to drinking bottled water.

This could be explained that in most developing countries in Asia, water sources are still not very well managed, unlike developed countries in Europe or America. Thus, boiled water from wells or river water is still common as the main source of daily drinking water [6–11]. This could result in higher risk factor of developing nephrolithiasis due to water consumption in Asian population than European or American population, where water source is more varied and healthy.

Another intriguing discovery in our study concerns smoking behaviors. Our analysis revealed an association between smoking and an increased risk of nephrolithiasis in Asian populations, a phenomenon which was also found in a previous systematic review [4,12]. Previous hypothesis regarding the increased prevalence of nephrolithiasis in smoking population is related to the increased vasopressin level, which can lead to low urine output and greater risk of kidney stone disease, while also increasing the release of reactive oxygen species in the kidney, accelerating the onset of chronic kidney disease [12]. A study from Soueidan et al. stated that smoking individuals had almost 9 times higher chance of experiencing urolithiasis compared to non-smoking individuals [13].

In addition, caffeine consumption has been identified as a potential risk factor for nephrolithiasis in European and American populations with odds ratio (OR) of 0.92 and 0.9, respectively, although these findings have not been replicated in all studies [14,15]. Another review of caffeine's effect on kidney stone formation signifies the fact that although it has a diuretic effect on enhancing urinary output, caffeine may slightly increase the stone risk index. On the other hand, three large cohorts have suggested that caffeine may play a preventive role in kidney stone disease. Previous retrospective and prospective studies have reported contradictory effects of caffeine on the risk of kidney stones [15, 16]. Daily energy drink and black tea consumption are also mentioned as significant factors of developing nephrolithiasis, although only study by Alblowi et al. stated about those two factors [11].

This again could be the highlight of this study, that in European and American population, metabolic problem such as higher body mass index is one of the main factor of developed nephrolithiasis [14,15]. This could be caused by multifactorial factors, but mainly due to high-fat dietary habits of European and American population, in contrary to dietary habits in developing countries [17–19].

Other potential risk factors included daily energy drink consumption, black tea consumption, and certain medications such as Losartan and statins, albeit based on a single study [17]. These inconsistencies demonstrate the need for additional rigorous research to validate and quantify the risks associated with these factors. Intriguingly, our research also suggested the possible function of external factors like psychological and psychosocial stress. However, the current literature lacks substantial evidence to support these associations, indicating an additional potential area for future study.

The limitations of this study are the number and the nature of the included studies. There were only 11 studies which were included in this systematic review, none of which reviewed all the risk factors discussed in this article. Moreover, all of the studies were either case control or cross-sectional study. Although studies with higher levels of evidence were rather tedious and time consuming, the conclusion drawn in this review was based on studies with lower levels of evidence, thus it should be carefully interpreted. Another limitation is that we did not analyze the correlation of associated risk factors with the stone type. Thus, this could be analyzed in future studies regarding the risk factor of urolithiasis.

## Conclusion

Many factors are directly or indirectly associated with the occurrence of nephrolithiasis in an individual. In this study, we figured out that two of the significant risk factors are family history and water consumption habits. Another notable risk factor that is mainly discussed is dietary habit, although this was not broadly discussed across studies.

The findings of this systematic review and meta-analysis will provide valuable insights into the risk factors of nephrolithiasis and may inform the development of preventive strategies for the management of this condition. Overall, these findings highlight the importance of considering regional differences in risk factors for nephrolithiasis when developing preventive measures and treatment strategies. Further research is needed to better understand the complex interplay between genetic, environmental, and lifestyle factors that contribute to the development of kidney stones in different populations.

## Abbreviation list

N/A: Not available
OR: Odds ratio
PRISMA: Preferred Reporting Items of Systematic reviews and Meta-Analysis
SD: Standard deviation
